# A Larger Ecology of Family Sexuality Communication: Extended Family Perspectives on Relationships, Sexual Orientation, and Positive Aspects of Sex

**DOI:** 10.3390/ijerph17031057

**Published:** 2020-02-07

**Authors:** Jennifer M. Grossman, Anmol Nagar, Linda Charmaraman, Amanda M. Richer

**Affiliations:** Wellesley Centers for Women, Wellesley College, Wellesley, MA 02481, USA; anagar@wellesley.edu (A.N.); lcharmar@wellesley.edu (L.C.); aricher@wellesley.edu (A.M.R.)

**Keywords:** teenage reproductive health, extended family, family sexuality communication, adolescent health

## Abstract

Extended family can be a resource for conversations about sex, but extended family perspectives have been largely left out of existing research. The present study investigates how extended family, such as aunts and uncles, siblings and cousins, perceive communication with teens in their families about sex. A thematic analysis was conducted with data from interviews in the U.S. with 39 extended family members, primarily siblings, who reported talk with teens in their families about sex. The analyses identified one theme focused on perspectives surrounding what is most important for teens to know about sex and relationships and seven themes focused on the content of conversations with teens about sex. The most prevalent content areas were: Healthy and Unhealthy Relationships (87%), Sexual Orientation (82%), Sexual Behavior (82%), and Protection (74%). The findings highlight extended family members’ unique roles in supporting the sexual health of teens in their families, which include providing information and support about issues other family members may not address, such as sexual orientation and the positive aspects of sex. The findings suggest the need to include extended family in sex education interventions to reflect the broader ecology of teens’ family relationships and access an underutilized resource for teens’ sexual health.

## 1. Introduction

Communication with parents about sex can support teens’ health through delayed sex [1] and more frequent safer sex behaviors [2]. However, teens and their parents are often reluctant to talk with each other about sex, and only half of teens report talk with their parents about this topic [3]. Extended family members often serve as resources for teens’ communication about sex [4,5] and initial research suggests that this communication may be protective against adolescents’ risky sexual behavior [4]. Despite these findings, both sexuality communication research and practice largely focus on teen–parent relationships. Extended family members’ perspectives are important, as they can help to understand how extended family support can complement teens’ communication with parents or address gaps when parents and teens don’t talk with each other about sex and relationships.

Conversations about sex and relationships provide a way for families to share sexual values with teens [6,7]. Sexual socialization theory explains a process through which individuals gain understanding about ideas, beliefs and values related to sex [8]. Parents are often seen as the primary source of sexual socialization messages for teens. However, teens also identify extended family as key sources of sexual information and support [5], suggesting the need to explore extended family roles in teens’ sexual socialization. Other sources, such as media, can also shape teens’ sexual socialization [9,10], but this exploration is largely beyond the scope of this study. 

Research shows that many teens talk with extended family about sex. A recent quantitative study of family communication about sex among diverse teens found that almost half of teens (46%) reported talking to an extended family member about sex, with the most sexuality communication reported with older sisters (24%), older female cousins (21%), older brothers (16%), and aunts (13%) [4]. Stepmothers have also been found to be sources of support for young people’s sexuality communication [11]. A study of teens’ perceptions of family communication about sex found that teens were more likely to describe extended family than parents as sharing valuable life experiences and being easy to talk to about sex [12]. A qualitative study of African American teens found that the largest variety of messages about dating and relationships came from mothers and siblings, followed by fathers, aunts/uncles and cousins [5]. A survey study of parent and grandparent caregivers found that grandparents were more interested than parents in having open conversations with teens about sex [13], although other studies suggest that few teens identify grandparents as a key source of sexuality communication [4]. 

Some studies explore the content of teens’ conversations with parents and extended family about sex. A qualitative study found that teens were more likely to report that parents, when compared to extended family, shared messages about delaying sex and avoiding teen pregnancy, but reported similar communications, when compared to extended family members, about protection, staying focused in school, and being careful in relationships [12]. A frequent gap in teen–parent communication about sex relates to sexual orientation. Studies show that less than half of parents talk with their teens about sexual orientation [14,15]. Parents’ communication with teens often includes assumptions of heterosexuality, such as only discussing potential partners of the opposite gender, rather than addressing sexual orientation directly [16,17]. However, a more recent study suggests that parents share more positive than negative messages with teens about sexual orientation [17]. Studies suggest that lesbian, gay, bisexual, transgender or queer (LGBTQ) teens often talk about their sexual orientation with their siblings [18,19], who are frequently seen as a source of support in the context of this disclosure [20,21]. Other female extended family members also serve as resources for LGBTQ teens’ communication about sexual orientation [18]. Research is needed to further explore the content of extended family communication with teens about sex, particularly regarding sexual orientation.

Communication with extended family might become particularly important as teens begin to explore relationships and sexuality. Teens may avoid talk with their parents about sex based on concerns that parents will disapprove of their sexual choices or worry about their sexual behaviors [11,22]. Extended family may provide a less judgmental alternative for teens to talk about sex and relationships and an opportunity to ask questions about sexual issues [22]. Furthermore, parents often focus conversations about sex on delaying sex, which is protective for teens’ initiation of sex [1], but is less relevant for sexually active teens.

The current study extends existing research by investigating extended family members’ perspectives on what they think teens should know about sex and the content they discuss with teens related to sex and relationships. We define an extended family to include the larger family context of non-parental sexuality communication: grandparents, uncles, aunts, older cousins and siblings. We include siblings as extended family because studies show that siblings play more of a peer role in talking with teens about sex than parents do [5,23] and teens’ communication about sex is similar with siblings and cousins [5,22]. However, since the research suggests that siblings play a unique and important role in teens’ sexuality communication [24,25], we include a preliminary assessment of whether sibling participants talk with teens about topics related to sex and relationships compared to non-sibling participants. This study can help to understand how extended family members fit into the larger family support system for sexuality communication with teens.

## 2. Materials and Methods

### 2.1. Recruitment and Participants

The data for this study came from 39 extended family members who identified a high school-aged teen in their families with whom they talk about sex and relationships. All participants gave their informed consent for participation in the study. Each participant was offered a $25 gift card to Amazon, Target or CVS in appreciation of their participation in the study. The study was conducted in accordance with the Declaration of Helsinki, and the protocol was approved by the Wellesley College Institutional Review Board (19 December 2016). This convenience sample was recruited through local schools and organizations, social media and national sites such as Amazon, Mechanical Turk (MTurk) and Facebook. To determine whether a person qualified for interview participation, individuals were asked three questions in a screening survey: (1) Do you have a younger sibling, cousin, niece or nephew or grandchild in high school? (2) Do you talk with them about dating, sex, or relationships? (3) Are you 18 or older? If individuals answered “yes” to all three questions, they were provided with the consent information and invited to participate in the study. All participants were told that the purpose of the study was to understand how teens and their families talk about sex and relationships to better understand families’ experiences and support teens’ health. Participants were also told about the protection of their privacy and the costs and benefits of survey participation. 

Participants self-identified as 74% female, 21% male, and 5% transgender. Participants’ ages ranged from 18–50, with the largest group of participants (46%) at 20–25 years old. Participants identified their racial/ethnic backgrounds as White (43%), Hispanic/Latinx (23%), Black (23%), Biracial/multiracial (8%) and Asian American (3%). Half of participants (49%) completed some college, 46% completed college and 5% had high school or less education. Fifty-three percent identified as older sisters to the teens they talk with, 13% as older brothers, 13% as aunts, 8% as uncles, 8% as cousins, and 5% as siblings (two non-gender binary participants). Eighty-five percent of participants reported they “sometimes” or “often” talk with the teen in their family about sex or relationships. All participants described the teens they talked with as high-school aged.

### 2.2. Interview Protocols and Procedures

The interviews took approximately 45 min each and were conducted over the phone. Before starting the interview, all participants were reminded that the purpose of the study was to better understand how teens and their families talk about sex and relationships. Participants were reassured that it would be normal to feel a bit embarrassed or uncomfortable and that they could choose not to answer any questions. They were asked to create pseudonyms to protect their confidentiality; those pseudonyms are used in this article. The interviews were audio recorded, then later transcribed. To start the interview, participants were asked to describe their relationship with the teen they identified in the screening survey and report how often they talked with the teen about sex and relationships. They were asked about what they believed the teen needed to know about sex and relationships, “In your opinion, what are the most important things that [teens’ name] should know about sex and relationships? Tell me about that”. Participants were asked whether they talked with the teen in their family about the following topics related to sex and relationships: dating, healthy or unhealthy relationships, sexual behavior, protection from STIs, protection from pregnancy, teen pregnancy, sexual orientation, misconceptions about sex, sexual orientation, and positive aspects of sex. If they answered “yes” to having talked about a topic with the teen, they were prompted, “Tell me about a conversation you had about this topic”. Participants were also asked about their age, gender, racial/ethnic background and education. After the interviews, all participants were e-mailed a resource list with contact information for organizations supporting youth social, emotional, and sexual health.

### 2.3. Data Analysis

We used thematic analysis to analyze the content of these conversations by coding for overarching themes related to participants’ perceptions of talk with teens in their families about sex that came up in multiple responses [26]. The first and second authors summarized the preliminary reflections on each interview and, together, developed the initial descriptive and interpretive codes. We then coded the study data, reviewed, revised, and defined themes. Themes are not mutually exclusive, because a single response could be coded under more than one theme. We did a preliminary comparison of sibling versus non-sibling participants’ responses based on the percentage of participants from each group who discussed each theme. NVivo 10.0, manufactured by QSR International in Melbourne, Australia [27] was used to facilitate coding. To guard against investigator bias, the first and second authors conducted reliability checks by coding the same data, then comparing responses to address coding inconsistencies, resolve differences in the perceived meanings of themes, and recode data as needed. The interrater reliability was calculated using Miles and Huberman’s formula, in which reliability equals the number of agreements divided by the total of agreements and disagreements [28]. The intercoder reliability of 92% represented a high level of agreement between the two coders.

## 3. Results

### 3.1. Interviews

We identified 8 themes; one theme, Importance, focused on family members’ ideas about what they thought the teens should know about sex and relationships, and the remaining seven themes focused on the content of conversations. The most prevalent topics of conversation with teens described by extended family members were: Relationships (87%), Sexual Orientation (82%), Sexual Behavior (82%), and Protection (74%). Participants also described conversations with teens about Teen Pregnancy (69%), Misinformation about Sex (64%), and Positive Aspects of Sex (54%). See Table 1 for the percentages of extended family members’ reports of the themes and subthemes. In discussing many of these topics, participants described how they talked with the teens about personal experiences related to their own relationships, the teens’ relationships, and relationships of other family members or people they knew. During the interviews, participants also discussed their motivations to talk with teens about issues of sex and relationships. Based on these two threads of participants’ responses, we created two subthemes that were identified across multiple topic areas: (1) Personal Talk includes conversations between the participant and the teen about personal experiences and (2) Self-Reflection addresses when, during an interview, a participant discussed what motivated them to talk about the topic with the teen. 

### 3.2. Importance 

The theme, Importance, addresses what extended family members identify as the most important things they think teens in their families should know about sex and relationships. This theme gives context to participants’ descriptions of different topics discussed with teens and frames their priorities for what they wanted the teens to know about sex and relationships. Importance was coded regardless of whether the teen and relative discussed the topic. All participants (100%) answered this question. It has four sub themes: Safer Sex (51%), Consent (38%), Delaying Sex (18%), and Okay to Talk about Sex (26%). 

The Safer Sex subtheme includes talk with teens about the importance of using protection methods to avoid sexually transmitted infections (STIs) and teen pregnancy; for example, Sara talked about what she wants her sister to know about safer sex, “What I would want her specifically to know is that you can be safe and you can protect yourself from pregnancy and STDs. You can have a really loving, joyful, satisfying, and happy, caring relationship, while still protecting yourself”. Meghan described what she wants her niece to know about sex “I’m all about erring on the side of caution, trying to be as safe as possible… obviously using condoms or other kind of STI protection”. 

The Consent subtheme addresses the importance of a mutually and willingly consensual relationship and red flags if consent is not present, such as physical and emotional abuse, partner pressure to have sex, and domestic violence. Phil described conversations with his brother as a supplement to conventional sex education, because “I really think consent is number one. I think that he knows to wear a condom and that kind of thing. That sort of thing doesn’t worry me as much, because they teach it in school and he has a sense of it. But in terms of consent stuff, there really isn’t any sort of education about it and his friends don’t prioritize it in the same way”. Mary talked about the importance of consent. “My sister and I don’t want our brother to end up being this jerk that treats women like crap. Unfortunately, we have been examples to him in a lot of cases in which he’s learned what consent is and what consent should look like because we’ve been with people who haven’t understood that and we’ve made examples out of that to him”. 

The subtheme of Delaying Sex addresses waiting to become sexually active until the teen feels ready. For example, Rebecca described what she wants her sister to know about sex, “I would want her to know that she doesn’t have to feel pressure to do anything she doesn’t want to do. It’s okay to say no if you feel uncomfortable. I would want her to know that it’s okay to wait because boys are not going anywhere—they’ll be here forever”. Similarly, Ashley talked about her sister, “I know she kind of feels pressured and stuff into it. And I just want her to know that it’s okay to wait and she doesn’t need to—she doesn’t need to rush anything”. 

The last subtheme was Okay to Talk about Sex and includes extended family members’ emphasis on why it’s important to talk about sex and to ask trusted adults questions about sex despite taboos about these conversations. For example, Alex shared that he wants his younger brother to know that “he can talk to adults about it (sex) and not be afraid to bring something up or worry that it’s going to be uncomfortable”. Likewise, Aunt Nina described wanting her niece to not “feel like its taboo or like wrong or shameful to ask questions, because no one knows—no one is born knowing”. 

### 3.3. Relationships 

The theme Relationships was the most frequent topic of discussion reported by participants. It includes conversations between teens and extended family members regarding the components and qualities that make up healthy and unhealthy relationships. This theme was discussed by 87% (34/39) of participants. It included four subthemes: Healthy Relationships (47%), Unhealthy Relationships (67%), Personal Talk (54%), and Self-Reflection (67%). 

The Healthy Relationships subtheme includes discussions between the teen and the extended family member about what makes a healthy relationship, such as mutual and willing consent, trust between partners, and supporting your partner. Brother John discussed that, in a healthy relationship, it is important for partners to “spend time, be compassionate, patient, and do fun activities that they both enjoy”. Similarly, Kathleen described explaining to her younger sister that the “most effective thing you can do in a relationship is be as open as possible, even if it’s uncomfortable for you”. 

The subtheme of Unhealthy Relationships encompasses discussions about the characteristics of unhealthy relationships, such as being controlling, mean, or physically or emotionally abusive. For example, Jane told her brother that “It’s not healthy to scream at someone. If you love somebody, you shouldn’t be having shouting matches regularly. You shouldn’t want to hit each other”. Jennifer described to her cousin what an unhealthy relationship looks like, “I tell her that the things that are not healthy in a relationship is like being with someone that’s abusive and that always knocks you down verbally, physically, whatever. You’ve got to watch out for that, because that’s not a healthy kind of relationship”. 

Participants described Personal Talk with teens about their experiences. For example, Maria described a conversation with her nephew in which she “asked him how his girlfriend is doing, he tells me some stories about the last time they hung out. Then I asked him if she’s pressuring him into doing something he’s uncomfortable with, to make sure he’s not pressuring her to do anything”. Seven described a conversation with her younger sister “explaining to her my first time, a situation where I felt like—I mean like afterwards—I realized I was definitely coerced”. 

Participants also described Self-Reflection during the interviews. Anna J described discussing relationships with her cousin, “We come from a family that had a lot of physical abuse growing up in terms of partners. So growing up having been around that, I really wanted her to know it is not healthy”. Sara talked about how her past relationship informed how she talked with her sister, “I’ve talked to her about if someone seems like they’re controlling. I just know because I’ve been in that situation personally, where I kind of saw some abuse flags coming and I got out of that relationship really quickly. And so I did kind of warn her, ‘If someone tries to keep you from seeing your friends, you know, that’s really something that you should be worried about’”.

### 3.4. Sexual Orientation 

The second most frequently discussed theme, Sexual Orientation, includes conversations between extended family members and teens regarding LGBTQ issues as well as extended family members’ reflections on how their own or others’ experiences shaped their conversations with teens about this topic. Eighty-two percent (32/39) of participants discussed this theme, which included four subthemes: Openness and Support (62%), Gender Identity (26%), Personal Talk (67%), and Self-Reflection (64%). 

The subtheme Openness and Support includes the extended family member expressing support and acceptance for LGBTQ identities whether or not the teen or the extended family member identifies as LGTBQ. For example, Jose described a conversation with his niece that reassured her that if she identified as a sexual minority, he would support her, “I had that conversation about orientation because she seemed to have her identity pretty well figured out, but that can change, especially in college years… I wanted to make sure that she understood that I was open to her feeling differently and maybe talking about it possibly, you know? And not just being a heterosexual female”. Alex described a conversation with his brother, in which he provided advice regarding sexual identity and resisting pressure to assign labels prematurely, “He’s talked to me about ‘I don’t know if I like girls or boys’. And I told him, you know, ‘Well you don’t have to know. In fact, you never have to decide. You can just be interested in whoever you’re interested in and that doesn’t mean you have to pick boys or pick girls’”.

The subtheme Gender Identity describes talk with the teen about defining or exploring gender identity/trans issues. At times, these conversations were in response to a family member dealing with a gender identity issue, such as when Maria gave an example of a time when she discussed this topic with her nephew, “Recently a member of our family came out as transgender. We had to talk about what that means and how it affects the person and the family. It was me doing a lot of talking and him asking a question or two, mostly asking for me to explain stuff to him like, ‘What do you mean?’”. Alex recalls a conversation with his sister in which he felt honored to have the opportunity to learn more about his sister’s non-binary-identifying community, “So I asked her questions like how do I refer to her friends, what are their favorite pronouns, etcetera. I thought that was a real bonding moment, because she was able to tell something to me that meant something to her. But it also served as a heads up (from the teen) of, ‘Hey, I’m queer’”.

Participants described Personal Talk with the teens. Rachel discussed conversations with her sister about how she identified with her sister’s struggle of being out during adolescence and the accompanying sense of alienation, “We’ll often talk about what it’s like to be… out in middle and high school because I was out in high school and she’s been out in middle school. We’ll talk about … what it’s like to… be in health class when none of the material being presented is relevant to you”. Amy talked about a conversation with her sister about appropriate pronoun usage with her sister’s non-binary friend, “… she doesn’t know if she’s transitioning or not. She was just asking me how to use the right pronouns and stuff. She was asking me, ‘How should I ask this girl? What’s offensive, what’s not offensive? I just said, ‘Well, you’re her friend, so of course you would be interested. You should just ask her like, ‘What pronouns do you want me to use? I don’t think they would get offended by that.’ And she was like, ‘Oh okay’”.

Participants shared Self-Reflections related to their conversations with teens about LGBTQ issues. For example, Alex described why he talked with his brother about sexual orientation, “Of the kids in my family, I’m like the only one who’s gay—or at least out as gay. So when I talk to him, I make sure that he knows that he can talk to me about his sexual orientation if he wants to. I’ll try to kind of keep that door open with him”. Another example of one’s family context shaping conversations about sexual orientation was when Kathleen commented about her own and her younger brother’s family experiences, “Our parents are gay and that was always something our parents would bring up and I openly discussed about myself when I was in college with them. And the kids in our family, even my cousins and stuff, have always like had LGBT friends. So that is just sort of something that comes up naturally”. 

### 3.5. Sexual Behavior 

The theme Sexual Behavior includes any substantive talk between extended family and teens about sexual behavior, including teens’ readiness for sex, teens’ or extended family member’s sexual experiences, and extended family feedback on teens’ sexual behavior and decision making. This theme was discussed by 82% (32/39) of participants. We identified three subthemes: Give Advice (46%), Personal Talk (33%), and Questions about Sex (31%). 

The subtheme, Give Advice, includes extended family members’ suggestions for teens about having sex, often emphasizing that the teen should decide when they’re ready and not be pressured to have sex. Many conversations started with teens’ questions about how they would know if they’re ready to have sex, or the relative recommending that teens wait to have sex. For example, Ashley recalls telling her younger sister “that she wants to be like 100% sure, because when she does lose her virginity, like there’s no taking it back. When she’s ready, just make sure it is the guy”. Similarly, Phil described telling his younger brother that “there’s nothing un-masculine or anything about not wanting to have sex this early on”. 

Participants described Personal Talk with teens about sexual behavior. Anna J talked with her cousin about “that journey that I had to go through to know that I wanted to have sex with my partner the first time I did have sex with him, and how I knew I was ready”. These conversations often included teens’ questions about sex, such as what to expect from sex, or how they feel about sex. Elizabeth recalled that her sister “called (her) after the first time and just said, ‘Do I need to take Plan B? Should I be worried about the fact that we did have sex or that I’m sore or anything like that?’”. 

### 3.6. Protection Methods 

The theme Protection Methods includes extended family members’ conversations with teens about pregnancy and STI prevention methods. Seventy-four percent (29/39) of participants discussed this theme and it included four subthemes: Use Protection (56%), STI Prevention (44%), Pregnancy Prevention (41%), and Access to Protection (26%). 

The subtheme Use Protection was applied when relatives described advising the teen to use protection or asking whether the teen was using protection. Jennifer cautioned her cousin, saying that “If you’re going to engage in something like that, make sure you always use protection no matter what, even if the guy says, ‘No, it’ll be okay.’ If he says that, then just back away from him and don’t talk to him”. John described checking in on his younger brother about his girlfriend regarding whether “he’s been using condoms lately”. 

The subtheme STI Prevention references condoms or other ways (e.g., delaying sex) to protect against sexually transmitted infections. Peter told his brother “there are lots of things and some are curable, but also there are diseases…that you’re going to have more or less indefinitely”. Likewise, Lucy described how she and her sisters made a slideshow for their younger brother “about the different sexually transmitted diseases and infections...to get the point across of why I buy (him) condoms every month”. 

The subtheme Pregnancy Prevention references condoms and other methods to protect against pregnancy. Alex talked to his sister about condoms “‘You may want to go with the spermicidal ones if you’re hooking up with a guy like you haven’t known too long. And even you were, it’s still a good idea of try to get the higher quality ones that are less known for ripping. Don’t ever store them in your pockets. Don’t use double ones’”. Maria addressed the theme in a humorous way with her sister, reflecting that “every time we’re listening to music, like we’ll joke about rubbers and stuff. Like it’ll be like, ‘Wrap it up,’ you know, because it’s a song or whatever and it’s talking about, you know, having sex. You’re not trying to get pregnant and you’re not trying to get anybody else pregnant”. 

The final subtheme is Access to Protection and includes extended family members and teens discussing where to get protection and/or the extended family member offering to get protection for the teen. Mary recalled telling her younger brother “If you ever need more condoms, just like let me know and I can get you some”. Alternatively, Ava directed her nephew to where he could get condoms, “You’ve got to make sure you use condoms. You can get them for cheap at the convenience store, but most schools nowadays even have them for free or the doctors even sometimes have them for free. So don’t ever feel like you can’t access them. They’re cheap, accessible, and it saves you a lot of worry later—later on”. 

### 3.7. Teen Pregnancy 

The theme Teen Pregnancy includes conversations between extended family members and teens regarding teen pregnancy or parenthood, as well potential teen pregnancy (scares). It was discussed by 69% (29/39) of participants and included three subthemes: Give Advice (38%), Personal Talk (31%), and Self-Reflection (44%). 

The subtheme Give Advice focuses on advice from the extended family member to the teen about avoiding teen pregnancy through methods such as abstinence or birth control. Anna J talked about a conversation with her cousin “she asked ‘Why can’t I be a teen mom? And why’s everybody stressing it?’ I was like, ‘Well, you haven’t fully developed yet in any way. Not to say that, ‘Oh my goodness, teen moms are horrible,’ but it is to say that you don’t finish developing and you don’t finish learning who you are before you have a child, and what if you didn’t want kids? Or what if you’re someone who doesn’t want children and you don’t even know that about yourself yet?’”. Megan described the advice she gave her niece, “The first time she had sex, she thought she was pregnant and had nobody to talk to. First thing we did was figure out if she was or not—which she wasn’t. But then I just told her that it’s the last thing she needs right now. ‘I understand if you’re going to do it, you’re gonna. I’m not going to tell her not to have sex or anything like that. Just be more careful… It’s not as glamorous as they make it on TV’”. 

Personal Talk included participants’ talk with teens about their own experiences of teen pregnancy or pregnancy scares or those of other people they knew. Gerald talked with his niece about her pregnancy scare, and “told her like, ‘Hey, these things happen. It was a mistake.’ And I asked her if she regretted it and she said, ‘Yes.’ And I was like, ‘What would you do different?’ And she was like, ‘Well, I wouldn’t do it’”. Jenny Reyes described a conversation with her younger brother, “I was pregnant when I was a teen, so he remembers a little bit of the struggle. He asked, ‘how hard is it to like keep up with school while being a parent?’ I explained to him, everything is harder but not impossible. Like a kid doesn’t really cut down your wings. You can still do a lot of things’”.

Self-Reflection includes participants’ discussions during the interview about personal experiences with teen pregnancy. Ava reflected on how her own experience as a young parent informs her conversations with her nephew, “I have a daughter who’s one year old. I got pregnant at 19 and so it was definitely a journey. I’m so glad I have my daughter and she’s such a blessing, but it was definitely tough. And you know, I don’t want him to go through that same situation until he’s ready, you know?” Shawn talked about how her family history shapes her conversations with her nephew, “His sister had a baby at 16, his mom had a baby at 16, and we’re trying to break the generational curse. So, wrap it up. I mean it’s that simple. If I’ve got to send you $10 for ear buds, who’s going to pay for the Pampers?”

### 3.8. Misinformation about Sex 

The theme Misinformation about Sex includes conversations where relatives talked with teens about sources of misinformation, myths, or inadequate information about sex and clarified potentially incorrect information (specifically, friends and media/porn were referenced). This topic was discussed by 64% of participants (25/39) and included two subthemes: Sources of Misinformation (49%) and Give Advice (31%). 

The subtheme Sources of Misinformation includes when teens or extended family members talked about misconceptions about sex conveyed by community, media/social media, school and faith-based organizations and peer sources. For example, Tina described talking with her brother “earlier this week [we] talked about pornography and how that’s a really bad place to get information about sex”. Elizabeth mentioned a peer source of misinformation to her sister, “I know that her one friend was pressuring her to take Plan B after she had sex, even though she was on birth control. And I definitely had to give her some education on why she shouldn’t take it”. 

The subtheme Give Advice includes feedback from extended family members advising teens to be educated about where they get their information from and to be critical of dubious information they may encounter. For example, Seven told her sister, “how sex on TV is not real and they exaggerate certain facial expressions and like sounds and stuff. And how it shouldn’t be a model of what sex should be”. Shawn told her nephew “I think your mom was pulling out when she had your sister at 16, so you might not want to follow her advice”. 

### 3.9. Positive Aspects about Sex

The theme Positive Aspects about Sex includes discussions about sex in a positive light. For example, saying that it “brings you closer together” or referencing the physical pleasure derived from it. Fifty-four percent of participants (21/39) discussed the positive aspects of sex in their conversations with teens. For example, Meghan described sex to her niece as “a thing that’s pleasurable, it is something to be enjoyed. And it’s great if you’re in a loving relationship, but it’s okay if you’re not, too”. Similarly, Sara shared with her sister, “‘Sex is really positive if you’re comfortable and happy with the person. You don’t need to be worried and as long as you’re safe and you protect yourself, it’s really a joyful thing.’ I think she sees it as just like a scary thing that will happen to her inevitably. And I’m like, ‘No, like it’s a thing that you’re in control. You set the boundaries that you’re comfortable with, and as long as you work within those, you can be really joyous and happy about it’”. However, a few participants expressed discomfort with discussing positive aspects of sex or expressed concern that they might encourage teen’s sexual activity by talking about the positive aspects of sex. For example, Rachel described her apprehension about talking with her sister about this topic, “I don’t want to be even inadvertently telling my sister that maybe she should be sleeping with people. I feel like those pressures are around enough already”. 

### 3.10. Preliminary Comparison of Sibling and Non-Sibling Participants’ Responses

The initial comparisons suggested that siblings may be more likely to report talk about Sexual Orientation, while non-siblings were more likely to talk about Protection Methods, Teen Pregnancy and Misinformation about Sex. There were fewer differences between siblings and non-siblings in whether they reported talk about Relationships, Sexual Behavior and Positive Aspects about Sex. See Table 2 for the percentages of sibling and non-sibling reported themes. 

## 4. Discussion

This is one of the few studies that explores extended family members’ perspectives around sexuality communication with teens in their families, with the exception of studies of grandparents’ attitudes and approaches to family sexuality communication [13,29]. This study explores what extended family members view as important to discuss with teens, what topics they discuss and how they approach talk with teens about sex and relationships. It provides an opportunity to understand a largely unrecognized resource for teens’ sexual health which may complement parents’ roles in teens’ sexuality communication or fill gaps in teens’ sexuality communication resources. These findings provide a window into how extended family members may be uniquely positioned to support teens’ health. 

Extended family members’ descriptions of their conversations with teens highlight their engaged role in teens’ sexual socialization. Participants often addressed topics such as safer sex, STIs, sexual orientation and the positive aspects of sex, which parents and teens may hesitate to discuss with one another and are often left out of sex education programs. For example, while parents frequently focus their communication with teens on delaying sex [12,30], many participants in this study described conversations with teens about safer sex and STIs. Parents may avoid these conversations due to fears that communication about safer sex will encourage teens’ sexual behavior [12], while teens may be reluctant to talk with parents about sex and protection due to worries that parents will judge them or be disappointed in their behaviors [11,22]. Sexual orientation is another topic that parents may avoid discussing or see as taboo [31], as less than half of parents talk with their teens about sexual orientation [15,32]. In contrast with parents’ reluctance to talk with teens about this issue, 82% of participants reported talking with teens about this topic. Similarly, some participants discussed the importance of discussing issues of consent with teens, in part because they are often left out of sex education classes [33]. The prevalence of consent in these conversations may also reflect a growing awareness of this issue due to the #MeToo movement and the prominence of consent issues in the public sphere [34]. Discussions about media influences, particularly pornography, which may provide false or misleading ideas about sexuality, was another focus of extended family conversations with teens in this study. Parents may avoid talking with teens about this topic due to fear and discomfort around addressing it [35]. Extended family members’ engagement with teens on topics that are often missing from other sources of sexual information and may be perceived as uncomfortable or taboo for other family members to discuss suggests the potentially unique contribution of extended family to teens’ understanding of sex and relationships. 

In contrast to prior findings that parents often don’t talk with their teens about sexual orientation [14,15] and may pass on implicit messages with a heterosexual bias [16], participants in our sample expressed an overall willingness to be a positive and supportive resource for teens regarding sexual orientation. This ranged from expressing messages which countered shame or stigma toward their teen family member should they come out as LGBTQ, to conversations about questioning and non-binary identities in both hypothetical terms and from personal experience. It is difficult to conclude to what extent high levels of engagement and support regarding LGBTQ issues stems from a growing public acceptance of sexual minorities, qualities of extended family members (particularly from younger generations), such as greater openness to LGBTQ issues, and the unique aspects of our sample, which may be unusually LGBTQ-friendly compared to the general population. Regardless of a teen’s sexual orientation, their attitudes and beliefs about sexual minorities are shaped by sexual socialization processes both within and beyond the nuclear family, such as other family members, media, school, and cultural or religious groups [17]. Extended family may provide one of the few positive sources of sexual socialization on this issue. 

About half of participants discussed positive aspects of sex—the lowest response rate for any of the seven sexuality themes. The comparatively small number of participants who discussed this topic, as well as the concerns expressed by a few participants that talking about positive aspects of sex would encourage the teen to have sex, suggests that a focus on the positive aspects of sex may push the boundaries of comfort for extended family. However, the inclusion of the positive aspects of sex in some conversations with teens indicates that extended family may be more open than parents in talking with teens about sexual issues. Conversations about the positive aspects of sex may be health-promoting for teens. However, teen–parent sexuality communication largely focuses on the risks involved with sexual activity, leaving out discussions about the positive aspects of sex [36,37]. Ideas about relationships and sexual activity in adolescence can be carried over to adulthood [38]. Therefore, it is important that teens learn about the positive aspects of sex as well as its risks. 

A focus on personal talk emerged across many content areas. Personal experiences served as a point of reference and connection in participants’ conversations with teens. Some participants expressed a hope that teens would learn from personal examples and either make use of these instances as positive models for relationships or conversely be less likely to repeat participants’ or other family members’ past mistakes. Studies show that parents describe their own personal experiences as a way to discourage teens from teen pregnancy and HIV-related risk behavior [39,40,41]. This suggests ways in which parents and extended family may use similar approaches to talking with teens about sex. However, communication about personal experiences related to sex, relationships, and sexual orientation as a way to support teens’ health has not been explored among extended family. In the interviews, participants also reflected on how their own negative experiences motivated them to talk with teens about sex and relationships. For example, participants often described a desire to prevent teens from going through negative experiences based on their relationship histories or those of others in their families. These dual processes of reflection on their own experiences and talk with teens about these experiences show the importance of going beyond the assessment of topics of conversation to understand the motivations for and processes of these family conversations.

An initial exploration of the themes for sibling and non-sibling participants suggests similarities and differences for these two groups. The high frequency of siblings’ reported talk with teens about sexual orientation fits with prior research that shows siblings as a key source of family support for teens’ talk about sexual orientation [18,19] and LGBTQ teens’ disclosures about coming out [20,21]. It may be that the combination of a peer-like relationship with teens [5,23] and close family connections make siblings feel like a safe resource to discuss topics which teens may experience as taboo to share with other family members. The lower frequency of discussion about protection methods and teen pregnancy for siblings, when compared to non-siblings, suggests that non-sibling family members may act in a similar way to parents in addressing traditional topics related to avoiding risky sexual behavior. Siblings may also believe that teens already have this information and it, therefore, doesn’t need to be a key topic for discussion with teens. The small number of cousins in the study (4) is a limitation and did not allow for separate exploration of this group. Quantitative research would be useful to statistically compare teens’ sexuality communication with different types of extended family members and what drives the topics of these conversations. 

This sample was primarily made up of extended family members from the same generation as the teens (siblings and cousins), considered horizontal relationships, which often show high levels of mutual disclosure when compared to vertical ones, such as the kinds of relationships teens have with their parents [42]. Grafsky and colleagues suggested that aunts (the other highly represent sub-group in our sample) may also have a special role in teens’ sexuality communication, due to relationships that are both emotionally close and have the authority to give direction and advice [18]. The lack of grandparents in this study is a limitation, particularly given studies that suggest their involvement in sexuality communication with teens [13,29]. However, the younger generations of extended family members included in this study are similar in age and generation to those teens typically report as their primary extended family communication partners [4], and may serve a more differentiated role than extended family in general. Future studies would benefit from comparing communication across different types and generations of extended family. 

This study uses a convenience sample of extended family members who are highly engaged in sexuality communication with teens in their families, in order to access the perspectives of a group that is rarely included in studies of family sexuality communication. We expect that people who volunteered to join this study are more engaged and comfortable in talking about sexual issues than many extended family members. Finding ways to include less engaged extended family members would help to clarify how generalizable this study’s findings are to a broader population. In addition, it would be useful, in future studies, to pair teens’ perspectives with those of extended family members, especially given findings that parents and teens are often inconsistent in their reports of how they talk with each other about sex [43]. Given the high level of engagement of extended family in this sample in talking with teens about sexual orientation, it would be beneficial to explore the potential supportive roles for extended family in talking with LGBTQ teens about sexual issues. A paired study would also allow for teens to identify which extended family members are the most influential to them for family sexuality communication. Our inclusion of participants who identified as transgender makes a unique contribution, but future research would benefit from a sample with more men, to enable assessment of whether and how gender differences often found in teen–parent sexuality communication research [6,44] may apply to teens’ communication with extended family. Further, we did not ask demographic questions about the teens with whom extended family members talked about sex and relationships and, therefore, do not have information about their gender identities. This information would be useful in exploring gender differences in extended family–teen relationships. 

Most sex education programs that include family outreach focus on parents [4,45]. This study’s findings suggest that teens’ extended family may also be potential resources for their sexuality communication. Extended family outreach may be particularly valuable for teens who identify as LGBTQ. Topics that seem taboo and are less frequently discussed by parents may be areas where extended family can contribute to family conversations about sex, such as sexual orientation, safer sex, the positive aspects of sex, and pornography. Perhaps, rather than asking teens to identify a single family member they talk with about sex and relationships, educators or counselors could talk with teens about the larger set of family resources they have access to for conversations about sex and relationships, and explore possible variation in who they might talk to about different sexual topics. 

## 5. Conclusions

The high number of teens who talk with extended family about sex [4,5] and the associations of this communication with teens’ sexual behavior [4,46] suggest the need to understand extended family roles in sexuality communication. This study is one of the first to provide an in-depth exploration of extended family perspectives on family sexuality communication. These findings suggest that extended family members may engage with teens on topics that parents rarely discuss with their teens or see as taboo content for conversations, and that siblings may play a key role in this communication. This study shows that extended family members are highly invested in sexuality communication with the teens in their families. Their conversations are often personal and connected, and reflect close relationships with the teens in their families. More exploration is needed to understand how extended family members fit within the larger ecology of teens’ family sexual socialization and how this communication can support teens’ sexual health. 

## Figures and Tables

**Table 1:** Identified Themes and Subthemes of Conversations between Extended Family Participants and Teens (*N* = 39).

Theme	Participant Response (%)
Importance	100%
Safer Sex	51%
Consent	38%
Delaying Sex	18%
Okay to Talk about Sex	26%
Relationships	87%
Healthy Relationships	47%
Unhealthy Relationships	67%
Personal Talk	54%
Self- Reflection	67%
Sexual Orientation	82%
Openness and Support	62%
Gender Identity	26%
Personal Talk	67%
Self-Reflection	64%
Sexual Behavior	82%
Give Advice	46%
Personal Talk	33%
Protection Methods	74%
Use Protection	56%
Sexually transmitted infections (STI) Prevention	44%
Pregnancy Prevention	41%
Access to Protection	26%
Teen Pregnancy	69%
Give Advice	38%
Personal Talk	31%
Self-Reflection	44%
Misinformation about Sex	64%
Sources of Misinformation	49%
Give Advice	31%
Positive Aspects about Sex	54%

**Table 2:** Identified themes for sibling (*N* = 25) and non-sibling (*N* = 14) participants.

Theme	Sibling (%)	Non-Sibling (%)
Importance	100%	100%
Relationships	88%	79%
Sexual Orientation	88%	71%
Sexual Behavior	80%	86%
Protection Methods	52%	85%
Teen Pregnancy	60%	93%
Misinformation about Sex	60%	79%
Positive Aspects about Sex	52%	57%
